# Infectious Aortitis Following Peripheral Angiographic Catheterization

**DOI:** 10.7759/cureus.86869

**Published:** 2025-06-27

**Authors:** Öykü Zeynep Gerçek, Ahmet Gurdal

**Affiliations:** 1 Internal Medicine, Sisli Hamidiye Etfal Training and Research Hospital, Istanbul, TUR; 2 Cardiology, Sisli Hamidiye Etfal Training and Research Hospital, Istanbul, TUR

**Keywords:** false aneurysm, infectious aortitis, percutaneous coronary intervention complications, sepsis, staphylococcus lugdunensis

## Abstract

Infectious aortitis and mycotic aneurysms are rare but potentially fatal conditions that often present with nonspecific symptoms, delaying diagnosis and treatment. The growing use of endovascular interventions has introduced new pathways for infection, particularly in high-risk individuals with vascular implants.

We report the case of a 67-year-old man with coronary artery disease who developed persistent lower back pain and systemic symptoms following peripheral angiographic catheterization and covered iliac stent implantation. Blood cultures repeatedly grew *Staphylococcus lugdunensis (S. lugdunensis)*, a virulent coagulase-negative staphylococcus known for its biofilm-forming capacity. CT angiography and 18F-fluorodeoxyglucose (FDG) PET-CT revealed an infected aortic pseudoaneurysm with perivascular inflammation, consistent with infectious aortitis and device-related infection. Despite targeted intravenous antibiotics, bacteremia persisted, indicating failure of conservative management. Surgical intervention was planned but ultimately declined by the patient, who deteriorated and passed away shortly after discharge.

This case highlights a rare instance of *S. lugdunensis*-associated infectious aortitis arising from a covered iliac stent. The persistent bacteremia despite appropriate antibiotic therapy strongly suggests biofilm-mediated infection, underscoring the limitations of medical therapy alone in managing device-associated infections. PET-CT proved instrumental in detecting metabolically active infection when anatomical imaging was inconclusive.

Clinicians should maintain a high index of suspicion for infectious aortitis in patients with vascular implants presenting with systemic symptoms and persistent bacteremia. Early use of PET-CT and timely multidisciplinary evaluation are essential for diagnosis and management. This report adds to the limited literature on peripheral stent-associated infections, particularly those caused by *S. lugdunensis*, and underscores the need for heightened post-procedural vigilance in similar cases.

## Introduction

Aortitis, in a broad definition, is an inflammation of the aortic wall and is associated with significant morbidity and mortality, including the development of an aortic aneurysm, aortic wall rupture, or acute dissection [1]. Aortitis could be caused by both infectious and non-infectious etiologies. Non-infectious etiologies are more prevalent than infectious etiologies [2]. The main causes of non-infectious vasculitis involving the aorta include large vessel vasculitides, namely Takayasu arteritis and giant cell arteritis, as well as other systemic vasculitides such as Behçet’s and Cogan’s syndromes. Rheumatoid arthritis, IgG4-related disease, and spondyloarthropathies are other possible causes [3]. 

Although both infectious and non-infectious forms of aortitis are rare, non-infectious causes, such as large vessel vasculitides, are more frequently recognized. Infectious aortitis, on the other hand, remains underdiagnosed due to its subtle presentation and low prevalence. With the increasing use of endovascular interventions, however, awareness of this potentially life-threatening condition is becoming ever more critical.

Infectious causes can be attributed to many pathogens, including staphylococci, enterococci, and streptococci. Infection of the aortic arterial wall can occur in the setting of iatrogenic or traumatic arterial wall injuries or from the spread of an infectious extravascular source that can potentially be adjacent to the arterial wall. Bacteria can seed the arterial wall through bacteremia or septic emboli, often initiating infection at sites such as ulcerated atherosclerotic plaques within the vasa vasorum [4]. 

Risk factors for infections of the aorta include atherosclerotic plaques and pre-existing aneurysms; impaired immunity states such as diabetes, glucocorticoid treatment, post-transplant, and HIV infection; an antecedent infection, especially from the vicinity of the aorta, such as pneumonia or osteomyelitis; and aortic injury from penetrating traumatic injuries to iatrogenic injuries such as cardiothoracic surgery [5]. 

Percutaneous vascular interventions are increasingly utilized in response to the rising global burden of atherosclerotic peripheral arterial disease. Complications of this treatment modality include access site bleeding and hematomas, contrast-induced nephropathy, dissection, and thrombosis. Danetz et al. [6] reported the following complication rates after aortoiliac and femoropopliteal angioplasty in the literature: bleeding, 3.4%; embolization, 2.3%; stroke, 0.55%; false aneurysm, 0.5%; renal failure and myocardial infarction, 0.2% each; and arteriovenous fistula, 0.1%. Emergency surgical exploration was required in 2.0% of cases, and amputation in 0.2%; death occurred in 0.2% [7]. 

Infectious aortitis could be caused by a variety of microorganisms, with gram-positive bacteria such as *Staphylococcus*, *Enterococcus*, and *Streptococcus *accounting for the majority (around 60%) of cases of infectious aortitis. Higher mortality rates have been reported with *Salmonella *spp.

The terms infectious aortitis and mycotic aneurysm are often used interchangeably, but they represent distinct though related clinical entities. Infectious aortitis refers to inflammation of the aortic wall caused by microbial invasion, which may or may not be associated with aneurysm formation. A mycotic aneurysm, in contrast, describes a localized, infection-induced dilation of the arterial wall. Typically resulting from hematogenous seeding, direct extension, or procedural contamination. Mycotic aneurysms may arise as a complication of infectious aortitis or occur independently in the setting of infected vascular grafts or endovascular devices. 

Mycotic aneurysms are aneurysmatic dilations of the arterial wall, typically caused by an infection. They may result from bacteremic seeding of a previously damaged vessel (e.g., atherosclerosis), iatrogenic injury during procedures, local extension from adjacent infections (e.g., vertebral osteomyelitis), or septic emboli. Although rare, accounting for 0.7% to 3% of aortic aneurysms, their mortality is significant. In Western countries, common pathogens include *Staphylococcus aureus* (*S. aureus*), *Salmonella *spp., and *Pseudomonas aeruginosa *(*P. aeruginosa*). [8] 

Diagnosis can be challenging due to the nonspecific clinical presentation and the rarity of the condition, which many clinicians may never have encountered firsthand. The abnormalities in the physical exam may include fever, pain, localized back or abdominal pain, or a pulsating mass. Laboratory abnormalities usually pertain to elevated acute-phase reactants like elevated erythrocyte sedimentation rate (ESR), C-reactive protein (CRP), or leukocytosis. The clinical presentation is variable and nonspecific, and this can make its diagnosis difficult.

*Staphylococcus lugdunensis* (*S. lugdunensis*) is a coagulase-negative, Gram-positive coccus that colonizes moist skin areas such as the groin, axillae, and toes. Though less commonly encountered than *S. aureus*, it has been implicated in invasive infections, including skin and soft tissue infections, prosthetic valve endocarditis, and osteomyelitis. In a study by Arias et al., *S. lugdunensis* accounted for 0.42% of skin and soft tissue infections, with trauma, surgery, or skin disease identified as the mode of entry in 75% of cases. [9] 

Here, we present a rare case of infectious aortitis and mycotic aneurysm following peripheral angiographic catheterization, attributed to *S. lugdunensis*, highlighting the diagnostic challenges and management considerations in such a rare but serious iatrogenic entity.

## Case presentation

A 67-year-old male patient with a known history of coronary artery disease presented with progressively worsening lower back pain that began two months ago, shortly after undergoing concurrent coronary and peripheral angiography. The pain, described as radiating to the scrotum and bilateral inguinal region, was accompanied by chills and worsened at night. Two months prior, he had undergone percutaneous transluminal coronary angioplasty with stent placement in the left common iliac artery. 

With regard to the percutaneous arterial intervention procedure, the indication for the procedure was critical limb ischemia with rest pain for the treatment of a subtotal stenosis and an aneurysmatic segment in the left common iliac artery. Peripheral angiography performed through the left common femoral access demonstrated a subtotal stenosis and an aneurysmatic segment. Percutaneous intervention was decided to be performed due to the debilitating symptoms refractory to lifestyle modification and medical treatment. The lesion was wired with a J wire through retrograde access. The lesion was predilated with a 6.0x40 mm Mustang™ balloon dilatation catheter (Boston Scientific Corporation, Natick, MA). A 7.0x59 mm Advanta™ V12 covered peripheral stent (Getinge AB, Gothenburg, Sweden) was implanted and post dilated with a 9.0x28 mm balloon. The aneurysm and the stenosis were repaired.

Concurrent with the peripheral angiography, cardiac catheterization revealed 70% stenosis in the proximal left circumflex and distal right coronary arteries. Given the absence of cardiac symptoms, the coronary lesions were managed conservatively in accordance with current clinical guidelines. 

The patient’s initial postoperative course was uneventful, and he was discharged on dual antiplatelet therapy. No signs of infection were documented during the immediate follow-up period. However, he missed subsequent scheduled outpatient visits.

He was scheduled for additional stent placement in the right superficial femoral and right common iliac arteries. Approximately two weeks after discharge, that is, six weeks prior to his current presentation, he began experiencing lower back pain, which progressively worsened and was ultimately accompanied by chills and systemic symptoms. This led to his evaluation at the cardiology outpatient clinic. The scheduled stenting of the right superficial femoral and right common iliac arteries was postponed due to the patient’s worsening back pain, systemic symptoms, and emerging clinical suspicion of an underlying infectious process. 

On examination at presentation, he was febrile (37.2°C) with a heart rate of 98 beats per minute (bpm), a respiratory rate of 18 breaths/min, and blood pressure of 128/79 mmHg. There was no costovertebral angle or spinal tenderness, and musculoskeletal examination was unremarkable.

Given the concerning clinical picture, the patient was admitted for further diagnostic workup and management.

Laboratory investigations performed at admission revealed markedly elevated inflammatory markers, including CRP (133.77 mg/L), procalcitonin (2.1 µg/L), and ESR (73 mm/h), raising strong suspicion for an underlying infectious process. Mild leukocytosis (10.85 × 10⁹/L) with neutrophilic predominance (80.9%) and hypoalbuminemia (2.8 g/dL) were also noted.

Differential diagnoses included infectious aortitis, an infected abdominal aortic aneurysm, adjacent spondylodiscitis, or retroperitoneal hematoma. 

**Figure 1:**
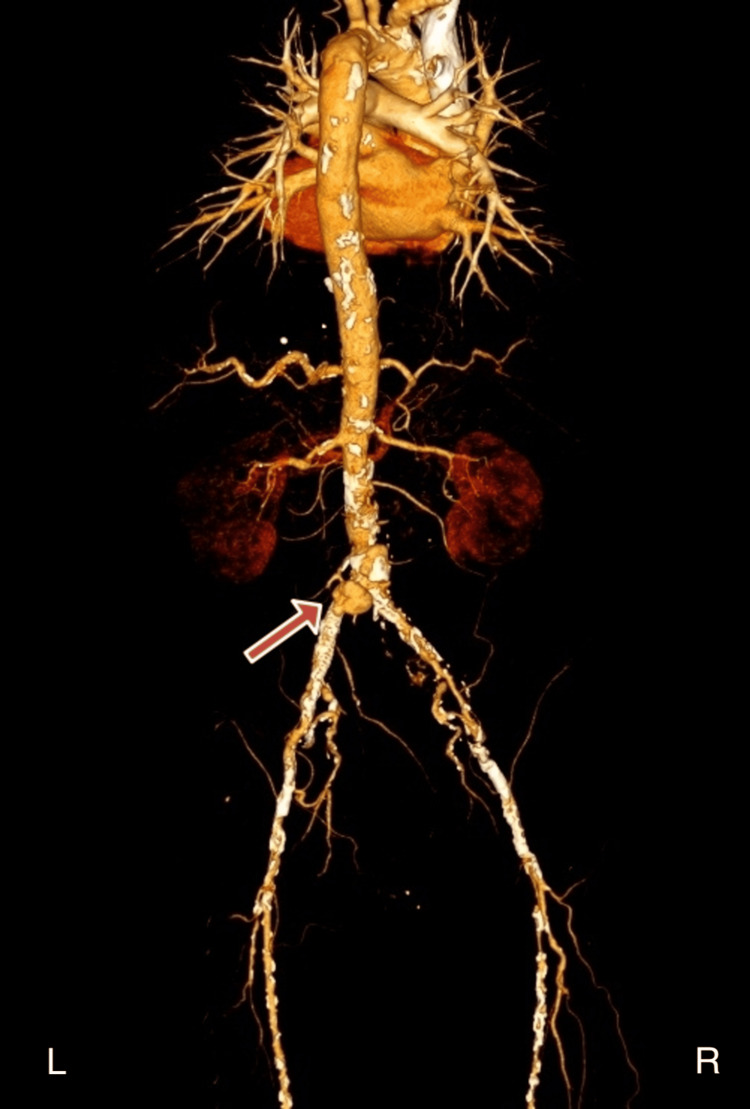
Abdominal aorta at the iliac bifurcation level and both common iliac arteries in their proximal segments Concentric perivascular hypodense soft tissue thickening is observed, reaching up to 6 mm at its thickest point, which was not seen in the previous examination. This may represent perivascular hematoma or aortitis, but infectious phlegmon is also a possibility in this patient. The stent is noted in the left common iliac artery. Contrast filling within the stent lumen is normal at this level; however, immediately at the iliac bifurcation level, near the origin of the left common iliac artery, a 21x12 mm contrast-filled pouch is seen posteriorly, suggestive of an endoleak with secondary pseudoaneurysm. L: left; R: right

Following this initial evaluation, two sets of blood cultures were obtained. An abdominal CT angiography was performed shortly thereafter on hospital day 1, which demonstrated perivascular concentric hypodense soft tissue thickening (up to 6 mm) surrounding the aortic bifurcation and common iliac arteries, which was absent on a scan performed six months earlier, findings suggestive of an infectious phlegmon. A contrast-filled pouch (21 × 12 mm) at the origin of the left common iliac artery, consistent with a contained pseudoaneurysm and possible endoleak, was also identified. No gas formation or abscess collection was observed. The remainder of the abdominal CT was within normal limits (Figure 1).

Empirical antimicrobial therapy with daptomycin and cefepime was initiated on the same day, based on clinical suspicion and preliminary imaging findings.

Two of the two blood cultures grew *S. lugdunensis*. Repeat cultures were done every 48 hours and remained positive in three consecutive culture sets. An echocardiogram was performed and was negative for intracardiac lesions or abscesses on the valve, septum, or papillary muscles. 

Antibiotic therapy was subsequently revised to cefazolin (3 × 2 g IV) and rifampicin (3 × 300 mg per oral (PO)), based on susceptibility testing that confirmed methicillin-sensitive *S. lugdunensis* (Table 1).

**Table 1:** Results of blood culture and antibiotic susceptibility tests MIC: minimum inhibitory concentration

Blood culture: Staphylococcus lugdunensis					
Antibiotic	MIC ( μg/mL)	Interpretation	Antibiotic	MIC ( μg/mL)	Interpretation
Benzylpenicillin	≤0.03	Sensitive	Cefoxitin	-	Sensitive
Clindamycin	≤0.12	Sensitive	Daptomycin	0.25	Sensitive
Erythromycin	≤0.25	Sensitive	Levofloxacin	≤0.12	Sensitive at high doses
Linezolid	1	Sensitive	Nitrofurantoin	≤16.0	Sensitive
Rifampicin	-	Sensitive	Tetracycline	≤1.0	Sensitive
Tigecycline	≤0.12	Sensitive	Trimethoprim sulfamethoxazole	≤10.0	Sensitive

**Figure 2:**
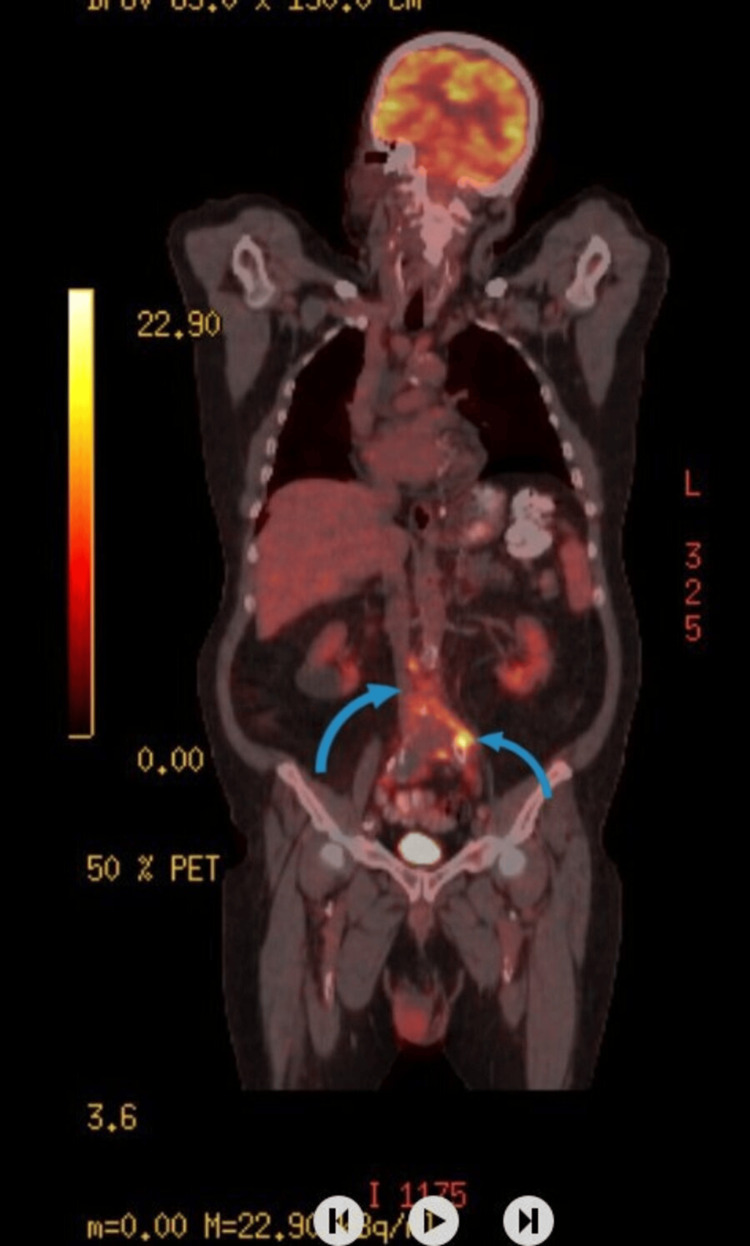
A full-body 18F-fluorodeoxyglucose (18F-FDG) PET-CT revealed increased perivascular thickening and FDG uptake (maximum standard unit value (SUVmax) 9.17) along the distal abdominal aorta and common iliac arteries, with inflammatory involvement of the anterior L4-L5 vertebrae and disc. A hypermetabolic (SUVmax 7.51) 10 mm inter-aortocaval lymph node was also detected.

In light of persistent symptoms and microbiological findings, a full-body 18F-fluorodeoxyglucose (FDG) PET-CT was promptly arranged and performed on hospital day 4 to further evaluate for vascular infection and to assess the possibility of adjacent osteomyelitis, given the patient's back pain and earlier differential diagnoses. The scan revealed increased FDG uptake (maximum standard unit value (SUVmax) 9.17) and perivascular soft tissue thickening along the distal abdominal aorta and bilateral common iliac arteries. No abnormal FDG uptake was observed in the vertebral bodies, disc space, or cardiac valves. A 10 mm hypermetabolic inter-aortocaval lymph node (SUVmax 7.51) was also noted and interpreted as reactive lymphadenopathy (Figure 2).

Despite one week of targeted intravenous antibiotic therapy, the patient’s symptoms persisted, and inflammatory markers showed a rising trend (CRP increased from 133.77 to 210.32 mg/L) (Table 2).

**Table 2:** Lab results overview: a comparison of key parameters at admission, Day 3, and Day 7 during antibiotic therapy

Variables	Day 1 (Admission)	Day 3	Day 7	Reference range
White blood cell (10^9/L)	11.66	13.11	12.44	4.5-10.5
Neutrophils (10^9/L)	9.78	11.37	10.36	1.78-5.38
Hemoglobin	10.3	11.1	9.6	13.0-17.0
Platelet (10^9/L)	292	338	247	150-400
Neutrophil-to-lymphocyte ratio	12.64	20.73	12.81	0.78-3.53
C-reactive protein (mg/L)	133.77	146.99	210.32	<5
Erythrocyte sedimentation rate (mm/h)	73	-	-	0-20
Procalcitonin (µg/L)	2.1	1.7	1.2	<0.5
Lactate (mmol/L)	1.79	-	2	<1
Albumin (g/dL)	2.8	-	-	3.5-5.5 (g/dL)
Creatinine (mg/dL)	0.86	0.96	0.91	0.70-1.20
Alanine transaminase (U/L)	15	16	29	<41

Given the inadequate response to medical therapy and persistent risk of progression, the patient was referred for cardiovascular surgical evaluation. The surgical plan included explantation of the infected stent graft with debridement and vascular reconstruction. He was transferred to the intensive care unit in preparation for the procedure; however, during preoperative stabilization, he developed hypotension, concerning for early septic shock. At this point, the patient declined further intervention and elected to leave the hospital against medical advice. The patient passed away shortly after discharge.

## Discussion

Diagnosing infectious aortitis, differentiating it from other conditions, and determining the appropriate treatment pose significant challenges for clinicians. Rapid diagnosis is critical due to the condition’s high mortality rate. A high index of suspicion is required, as patients often present with nonspecific symptoms, leaving clinicians to rely on findings such as fever, leukocytosis, and elevated inflammatory markers. In our case, differential diagnoses such as vertebral osteomyelitis, retroperitoneal abscess, and hematoma were considered, underscoring the diagnostic complexity. 

In this context, the combination of persistent lower back pain radiating to the inguinal region, systemic inflammatory response, and recent stent placement raised suspicion for infectious aortitis rather than more common post-endovascular complications such as access site hematomas, retroperitoneal hemorrhage, or localized pseudoaneurysm.

While the patient’s lower back pain was nonspecific and the physical exam provided no clear localization of infection, his recent percutaneous peripheral vascular intervention and stent placement raised clinical suspicion. Although vertebral osteomyelitis was considered early in the differential due to symptom location, PET-CT showed no abnormal uptake in the vertebrae or disc space, effectively ruling it out.

Infectious aortitis is rare due to the intrinsic resistance of the aortic wall to infection. It can develop through various mechanisms, including hematogenous seeding of a preexisting intimal injury, septic embolization, direct extension from a nearby infectious site, or bacterial inoculation [5]. However, identifying the primary source of infection remains difficult, with a definitive cause found in only about half of reported cases. In our patient, the infection was linked to a previously placed peripheral stent. Alternative sources of bacteremia, including skin infections, recent dental procedures, and urinary tract infections, were clinically considered but not identified.

Mycotic aortic aneurysms may result from hematogenous bacterial colonization of a preexisting aneurysm or spread from an adjacent infection. In rare cases, however, direct inoculation from vascular instrumentation or stent placement may serve as the primary source of infection. In our case, the infection appeared to originate from a covered, balloon-expandable iliac stent, an unusual but increasingly recognized iatrogenic pathway [10].

CT angiography remains the preferred initial imaging modality, offering both diagnostic clues, such as vessel wall thickening, periaortic soft tissue density, and fluid accumulation, and detailed anatomical mapping to guide surgical debridement or repair. The progressive inflammatory changes seen on imaging, along with persistent bacteremia, support the likelihood of biofilm-associated infection on the stent surface. In this context, 18F-FDG PET-CT served as a valuable adjunct, enhancing early detection by identifying metabolically active infection foci not always apparent on anatomical imaging. PET-CT offers functional insight into inflammatory activity, high-resolution imaging, and increased sensitivity in chronic or device-related infections, thus playing a complementary role in both diagnosis and management planning [11].

Diagnosis relies on blood cultures, though they yield positive results in only 50% to 82% of cases. The most commonly implicated pathogens are *S. aureus* and Gram-negative bacteria. In our case, *S. lugdunensis*, a rare but clinically significant organism, was identified. While classified as a coagulase-negative *Staphylococcus*, *S. lugdunensis* is notably more virulent than other species, such as *Staphylococcus epidermidis* (*S. epidermidis*) or *Staphylococcus saprophyticus* (*S. saprophyticus*). It exhibits pathogenic behavior more akin to *S. aureus*, with the ability to cause aggressive, deep-seated infections. It has been implicated in skin and soft tissue infections, native and prosthetic valve endocarditis, catheter-related bacteremia, prosthetic joint infections, osteomyelitis, and discitis. Its strong capacity for biofilm formation and adherence to foreign bodies likely contributed to the infected stent in our patient [12]. 

The persistence of bacteremia despite targeted intravenous antibiotic therapy was a critical finding in our case. Repeated positive blood cultures, particularly in the absence of a new source, strongly suggested the presence of a biofilm or infected foreign body, in this case, the covered stent. This underscored the limitations of conservative therapy in achieving source control and ultimately guided the decision to pursue surgical intervention. Persistent bacteremia in the context of device-related infections is a well-established indicator of treatment failure and should prompt early consideration of surgical management. 

The proposed procedure, explantation of the infected stent graft with debridement and in situ vascular reconstruction, was deemed high-risk due to the anatomical complexity and the patient’s overall condition. Although preparations for surgery were underway, the patient ultimately chose to leave the hospital against medical advice prior to the intervention.

Biofilm formation on the stent likely contributed to persistent *S. lugdunensis* bacteremia, as biofilms limit antibiotic penetration and shield bacteria from immune clearance. Rifampin was selected for its excellent biofilm penetration and synergistic bactericidal activity. While in vitro biofilm assays and molecular resistance testing were not available in this case, such methods may have offered additional insights into the organism’s biofilm-forming capacity, resistance gene expression, and the potential efficacy of alternative treatment strategies. Their integration into clinical decision-making could be particularly valuable in managing persistent device-related infections [13].

Although no guideline addresses infected peripheral stents directly, consensus statements on vascular graft and endograft infections consistently recommend prompt surgical removal and reconstruction once infection is identified. The 2016 American Heart Association (AHA) Scientific Statement on vascular graft infections advocates removal of infected prosthetic material to achieve source control [14]. 

Similarly, the European Society for Vascular Surgery (ESVS) 2020 guidelines recommend that in suspected endograft infection, complete graft explantation with in situ reconstruction should be performed without delay [15].

Given the high-risk nature of the proposed surgery and the patient's initial clinical stability, a short course of targeted intravenous antibiotics was initiated while preparing for surgical intervention. Once the diagnosis was established, cardiovascular surgical consultation was obtained without delay. Despite this multidisciplinary approach, the patient's rapid clinical deterioration ultimately precluded definitive operative management.

Treatment of infectious aortitis and mycotic aortic aneurysm typically involves broad-spectrum intravenous antibiotics, surgical excision of the infected segment, debridement of periaortic soft tissue, and either in situ or extra-anatomic reconstruction. In non-surgical candidates, especially those with high operative risk or poor prognosis, prolonged suppressive antibiotic therapy may be considered as a palliative or temporizing measure. Early surgical intervention is generally favored in patients with signs of sepsis, persistent bacteremia despite targeted therapy, enlarging pseudoaneurysm, or end-organ compromise. 

Given the increasing use of vascular stents and the challenge posed by biofilm-associated infections, especially with virulent organisms like *S. lugdunensis*, updated guidance on surveillance and management may be warranted, particularly in high-risk individuals, where closer post-procedural monitoring could improve early detection and outcomes.

A review of the literature revealed similar diagnostic challenges reported by other clinicians, yet also highlighted the unique aspects of our case. Huynh et al. [16] described a mycotic aneurysm caused by *S. lugdunensis* in a patient with a bioprosthetic valve and a history of tetralogy of Fallot. This remains the only well-documented case of *S. lugdunensis*-associated aortic pseudoaneurysm to date. However, no prior reports link *S. lugdunensis* to infectious aortitis following covered iliac stent implantation, nor do they describe biofilm-mediated infection in this specific context. A similar clinical trajectory was reported by Robinson et al. [17], who described an infected infrarenal aortic stent graft following transcaval embolization with glue and coil placement for a type II endoleak. While that case underscores the diagnostic complexity and clinical risk of post-procedural graft infections, it differs from ours in both etiology and anatomical site. To the best of our knowledge, the present case represents the first report of *S. lugdunensis*-associated infectious aortitis involving a peripheral covered stent.

## Conclusions

Infectious aortitis and mycotic aneurysms are rare yet life-threatening conditions, often challenging to diagnose due to their nonspecific presentations. Management typically requires prolonged intravenous antibiotics, surgical excision, or both. In our case, *S. lugdunensis*, a virulent and increasingly recognized pathogen, was identified through serial blood cultures, and CT angiography with 18F-FDG PET-CT supported the presence of an infected aortic pseudoaneurysm and device-related infection.

The infection occurred following the placement of a balloon-expandable, covered iliac stent, highlighting a novel iatrogenic pathway of direct inoculation and biofilm formation. Despite targeted antibiotic therapy, persistent bacteremia indicated failure of conservative treatment and the need for surgical source control, which could not be achieved due to the patient’s refusal. This case underscores the importance of early multidisciplinary intervention and the limitations of medical therapy alone in device-associated infections. It also illustrates that persistent bacteremia in the context of vascular implants should raise suspicion for biofilm-mediated infection, particularly with pathogens such as *S. lugdunensis*. Additionally, it highlights the diagnostic value of PET-CT in suspected vascular infections when anatomical imaging is inconclusive and calls for heightened vigilance regarding infection risk in the covered peripheral stent scenarios not yet well-characterized in the literature.
